# Tumor-Derived Soluble MICA Obstructs the NKG2D Pathway to Restrain NK Cytotoxicity

**DOI:** 10.14336/AD.2019.1017

**Published:** 2020-02-01

**Authors:** Qizhi Luo, Weiguang Luo, Quan Zhu, Hongjun Huang, Huiyun Peng, Rongjiao Liu, Min Xie, Shili Li, Ming Li, Xiaocui Hu, Yizhou Zou

**Affiliations:** ^1^Department of Immunology, Basic Medical School of Central South University, Changsha, Hunan, China.; ^2^Department of Physiology, University of Texas Southwestern Medical Center at Dallas, TX, USA; ^3^Cancer Hospital of Hunan, Xiangya Medical School, Central South University, Changsha, Hunan, China.

**Keywords:** hepatocellular carcinoma (HCC), NKG2D, soluble MICA, NKG2D receptor pathway, tumor immune escape

## Abstract

The natural killer group 2D (NKG2D) receptor and its ligands play important roles in immune surveillance. In this study, we observed that the average serum soluble MICA (sMICA) concentration of 174 hepatocellular carcinoma (HCC) patients was significantly higher than that in 80 healthy subjects (602.17 ± 338.15 vs. 72.26 ± 87.88 pg/ml, t = 3.107, P=0.002). The levels of serum sMICA in 44 HCC patients with initial levels above 400 pg/ml declined significantly after surgical removal of the liver cancer tissue (P<0.001). Moreover, the mean survival time of HCC patients who had sMICA above 400 pg/ml was significantly shorter than that HCC patients with lower sMICA levels (P<0.001). Using the reporter cell line (NKG2D-2B4) in which activation of the NKG2D receptor pathway results in GFP expression based on the stimulation of immobilized rMICA, we showed that the number of GFP-expressing cells decreased sharply in presence of sMICA. Upon adding sMICA, the release of cytokines IFN-γ, TNF-α, and IL-8 by NK cell line (NKL) under stimulation of immobilized rMICA was blocked. Using MICA-expressing cells as the target cells, we observed that about 80% of target cells were killed by NKL at E:T of 10:1, but in presence of sMICA^high^ serum of HCC patients, the dead target cells were reduced to 30.8%. Compared in presence of sMICA^low^ serum from HCC patients, there were 63.7% of target cells dead (p=0.043). Thus, our data suggested that sMICA obstructs the activation of NKG2D pathway to protect tumor cells from NK cell-mediated cytotoxicity.

Natural killer (NK) cells play an important role in immune surveillance for viruses and tumor cells in a way that is independent from antigen presentation by major histocompatibility complex (MHC) molecules [1, 2]. There are many inhibitory and activating receptors on the surface of NK cells; NKG2D [3], an important activated receptor, belongs to the type II transmembrane type C lectin-like receptor family [4-6]. Binding to NKG2D activates the NKG2D pathway and triggers cytotoxic activities of NK and CD8^+^ T cells against target cells [7]. The NKG2D receptor ligands include the major histcompatibility complex class I related chains A and B (MICA and MICB, respectively) and UL16 binding proteins 1-6 (ULBP1-6) [8]. MICA and MICB are expressed in gastrointestinal epithelial cells and fibroblasts at low levels under physiological conditions [9]. Upon infection or in the presence of a tumor, the expression of MICA and MICB increased [10]. We previously reported that human fibroblasts that overexpressed MICA were more sensitive to NK cells than normal fibroblasts [11]. Furthermore, we discovered that human cytomegalovirus infection leaded to a decrease in the expression of MICA on the fibroblast surface, which allowed these virally infected cells to resist NK cell-mediated cytotoxicity. The reduction of NK cell-mediate killing during viral infection and tumor genesis is associated with a decrease in levels of the NKG2D receptor and NKG2D receptor ligands [12].

MICA expression is regulated under stress conditions [13]. When MICA binds to the NKG2D receptor of NK cell, the associating membrane molecules DAP10 are phosphorylated to recruit and activate PI3K downstream pathway, resulting in MICA-expressing target cell killing upon NK cell activation [14-16]. Tumor cells have evolved a variety of immune escape mechanisms. Soluble NKG2D ligand molecules that block NKG2D activation were observed in patient with a variety of solid tumor types [17]. For example, tumor cells release soluble MICA (sMICA) by proteolytic shedding off from membrane-associated MICA [18, 19]. In osteosarcomas cells, it is MMP9 that hydrolyzes MICA [20]. In glioblastoma cell lines, ADAM10 and ADAM17 hydrolyze ULBP2 to form another soluble NKG2D receptor ligand [21]. Jiang et al. found that certain *MICA* gene polymorphisms are associated with survival in hepatocellular carcinoma (HCC) patients [22]. HCC tumor cells shed sMICA [23]. It has been proposed that sMICA can specifically bind to the NKG2D receptor without activating NKG2D-mediated signaling. Moreover, sMICA binding may prevent the NKG2D receptor from contacting NKG2D receptor ligands on the tumor cell surface, which may allow tumor immune escape [24].

In our previous study, we showed that a fusion protein composed of the MICA extracellular domain (MICA-ECD) and an anti-CD20 single-chain antibody (ScFv) induced NK cell-mediated killing of CD20^+^ tumor cells [25]. NKG2D and its ligands are involved in the development of prostate cancer, leukemia, melanoma, and other tumors [26]. Others have suggested the potential of immunotherapies based on the NKG2D pathway [27]. Here we investigated how serum sMICA levels in HCC patients are correlated with survival and studied how sMICA influences the activation of the NKG2D pathway using an NKG2D receptor reporter cell line.

## MATERIALS AND METHODS

### HCC patient samples

Blood samples were drawn from 174 patients diagnosed with HCC in Hunan Cancer Hospital from January 2017 to December 2018. The cohort included 137 males and 37 females with an average age of 55 ± 13 years. All HCC patients were diagnosed based on examination of tissue by pathologists and other clinic diagnosis. The control group included 80 healthy volunteers (62 males and 18 females) with an average age of 56 ± 12 years. All participants in this study provided informed consent. This study was approved by the ethics committee of Hunan Cancer Hospital and conducted in strict accordance with the requirements of the ethics committee.

### Cells and cell culture

Human fibroblasts cell (HFC), T hybridoma 2B4 cells, and the human NKG2D reporter cells (NKG2D-2B4 cells), which have been prepared as our previous work [25], were cultured in RPMI-1640 medium with 10% FBS at 37 °C in 5% CO_2_. The NKG2D reporter cells were constructed NKG2D receptor on 2B4 cells containing a nuclear factor of activated T cells (NFAT)-responsive green fluorescent protein (GFP) reporter gene [28]. The engagement of the NKG2D receptor induces the activation of NFAT and expression of GFP. The NK cell line (NKL, provided by M. J. Robertson, Indiana University School of Medicine, Indianapolis, IN) was cultured in RPMI-1640 medium with 15% FBS and 10 ng/mL of recombinant IL-2 (Sino Biological Inc.) at 37 °C in 5% CO_2_. MICA stable expressed human fibroblast cells (MICA^+^HFC) were prepared by transfection of the vector pCDNA3.1 (-) inserting with the cDNA encoding full-length MICA and were selected with 100 μg/ml G418 in culture medium.

### Preparation of recombinant MICA and NKG2D-Ig

HEK293T cells (1×10^6^ cells) were added to 10 ml DMEM, and the cells were cultured at 85%~90% confluence. At 1 hour before transfection, 5 ml of complete culture medium without antibiotics was added. Constructed vectors with codons of MICA*004 extracellular domains fused to a His-tag and vectors with codons of NKG2D extracellular domains fused to a human IgG-Fc and a His-tag were added, then culturing for 72 hours and collecting the culture media. The media were centrifuged at 10000 rpm for 5 minutes, and supernatant was collected and stored at -80 °C if needed, The supernatant contained the recombinant proteins of rMICA*004-His_6_ and NKG2D-Ig-His_6_ were dialyzed for 5 days against Tris-HCl buffer (pH 7.5). The buffers were changed with new one every 12 hours. After dialysis, NaCl and imidazole were added to final concentrations of 500mM and 20mM, respectively. Ni-NTA protein purification kit (pH=6.8-7.2, Konipa, Guangzhou, China) was used to purify the His-tag fusion proteins in solution according the instruction of manufactory.

### Sandwich ELISA to detect sMICA

Anti-MICA antibody (AMO1, Immatics; mouse IgG1, 5.0μg/ml) was coated to reaction wells (100μl per well) at 4°C over night, and after washed with PBS containing 0.05% Tween-20, 100μl the blocking buffer (PBS containing 5% BSA) were added and incubated at 37 °C for 2 h. Then after removal of blocking buffer, washed the well with washing buffer (PBS containing 0.05% Tween-20) for two times. Next, rMICA*004 was diluted to prepare eight standard solutions within the range of 100 to 6309 pg/ml. Standard solutions, serum samples were added into each well, respectively; after incubated at 37 °C for 2 hour, wells were washed with 200μl of washing buffer for four times. Then 100μl of MICA-specific monoclonal antibody (6B3) [29], mouse IgG2a, 1.0μg/ml diluted with PBS containing 3.75% BSA) was added to each well. After incubation at 37°C for 2h, wells were washed with washing buffer for four times, then, 100μl HRP-conjugated goat anti-mouse IgG2a antibodies (BD Biosciences) diluted with PBS containing 3.75% BSA at 1:10000 ratio was added to each well and incubated at 37 °C for 1h. After washed with washing buffer for four times, 100μl of chromic substrate (tetramethyl benzidine) was added to each well, and the reaction was terminated with 4N H_2_SO_4_ after incubation at 37°C for 15 min. The OD value was detected at the wavelength of 450 nm, and sMICA in serum was quantified by comparison to the standard curve generated by analysis of the recombinant protein.

### Flow cytometry

The level of GFP-expressing NKG2D-2B4 reporter cells was determined by flow cytometry. The rMICA and control BSA at 50μg/ml were added to each well of a 96-well plate in a 50 μl volume, respectively. To perform as the immobilized ligand of NKG2D receptor (immobilezed-rMICA) and allow rMICA adhere to the well, the soluble rMICA was added to wells and incubated at 37 °C for 4 h. After washed with PBS, the concentration of NKG2D-2B4 cells was adjusted to 4x10^5^ cells/ml with RPMI-1640 medium containing 10% FBS, and 200 μl of this cell suspension was added to each well. GFP generated under NKG2D receptor activation was detected by flow cytometry after incubation at 37 °C for 16 h. The degree of activation of the NKG2D signaling pathway was determined based on the percentage of GFP^+^ cells. Anti-NKG2D antibody (1:2000, Thermo Fisher Scientific) and anti-His tag antibody (1:1000, Bio-Rad) were used for detection of NKG2D expression and the binding of his-rMICA*004 to NKG2D receptors of cells.

Meanwhile, 2×10^6^ NKG2D-2B4 reporter cells were collected, centrifuged at 800 rpm for 5 min, washed with PBS containing 2% FBS, and then re-suspended in PBS. Subsequently, w6/32 (5 μg/ml, ATCC, USA) was added, anti-NKG2D antibody was added at 1:200, and anti-MICA antibody was added at 1:200 (antibody concentrations were 5 μg/ml). After incubation at 4 °C for 30 min, 2% FBS-PBS was added, and then anti-mouse IgG FITC monoclonal antibody (BD Pharmingen, 1:400) was added and incubated at 4 °C for 30 min. The cells were re-suspended with 500 µl 2% FBS-PBS and analyzed by flow cytometry (Athena dxp, Cytek Company).

### Absorption of sMICA from serum

50 µg/ml stock of anti-MICA specific monoclonal antibodies 6B3 (mAb-6B3) was coated to the protein A micro-magnet beads (Kangsheng Biotechnology, Guangzhou, China). After incubated at 37 °C for 8 h. The supernatant was discarded, and the pellet was washed with PBS three times, HCC patient serum, diluted 1:3 in PBS (pH7.5) was added into 6B3-beads prepared, and incubated at 37 °C for 2 h. Then, repeated above absorption procedure. Finally, the treated samples were collected and saved in -80 ? until use. In addition, the same serum sample was proceeded the sMICA absorption by uncoaded beads and used as control.

### NK cell cytotoxic activity

Target cells of human fibroblasts with or without stable expression of MICA*027 by viral transduction were collected by centrifugation at 800 rpm for 5 min. After washing with sterile PBS, cells were centrifuged at 800 rpm for 5 min. The target cells were suspended in sterile PBS to 1×10^6^ cells/ml. CFSE (Sigma) was added into the cells at a final concentration of 200 nM in PBS, and cells were stained at 37 °C for 20 min. After washing three times with PBS, the cells were transferred into wells of a 96-well plate at 5000 cells per well. NK cells were added to the wells with the target cells stained with CSFE at a ratio (E:T) of 1:1, 5:1, 10:1, and 20:1 in a final volume of 200 µl. After mixing, the cells were centrifuged at 800 rpm for 1 min and incubated at 37 °C for 4 h. After centrifugation at 2000 rpm for 2 min, the cells were collected and washed twice with PBS supplemented with 2% FBS, stained with 7-AAD (7-Amino Actinomycin D, BD Pharmingen^TM^) at room temperature for 10 minutes. In NKG2D pathway blocking assays, purifed rMICA diluted at concentrations among 0.1 ng/ml to 8.0ng/ml, sMICA^high^ sera from HCC patients (sMICA>400pg/ml, n=10), sMICA^low^ sera from HCC patients (sMICA ≤400 pg/ml, n=10), normal health serum and soluble NKG2D-Ig at concentration above 1.0 ug/ml were added to the mixture of target cells with NKL to block NKL killing, respectively. After mixing, the cells were centrifuged at 800 rpm for 1 min and incubated at 37 °C for 4 h. After centrifugation at 2000 rpm for 2 min, the cells were stained with 7-AAD. After washing with PBS, cells were fixed with 1% paraformaldehyde in 2% FBS and 0.1% NaN_3_ PBS buffer; the dead cells were analyzed by flow cytometry.

### NKG2D pathway activation and blocking

rMICA at range of concentration was added to wells of a 96-well plate, allowing rMICA to adhere to the well of plate and incubated at 37 °C for 8 hours. Un-coated rMICA in the solution was discarded. NKG2D-2B4 cells were added to each well at a range of concentrations. After the cells were incubated for 24 h at 37 °C, the percentage of fluorescent cells was determined by flow cytometry. The concentration which generated the maximal number of fluorescent cells (GFP^+^) was used in subsequent experiments.

For analysis of pathway activation and pathway inhibition in the presence of serum, rMICA at a final concentration of 50 µg/ml was coated to wells of a 96-well plate for NKG2D receptor activation. The solution was discarded after 8 h at 37 °C. Aliquots of 100 µl of NKG2D-2B4 cells at 1×10^6^ cells/ml were added to the tubes containing soluble rMICA, normal human sera, patient sera or soluble NKG2D-Ig fusion protein, and incubated at 37 °C for 2 h. Then aliquots of 100 µl of treated and untreated cells were added to wells of 96-well plates with pre-coated rMICA (immobilized rMICA). After mixed, the cells were incubated at 37 °C for 4 hours. The proportion of cells with fluorescence was detected under a fluorescence microscope and analyzed with flow cytometry (Athena dxp, Cytek Company).

### Cytokine detection

The cytokine Grp I group 17-plex kit (Bio-Rad) was used for this experiment. The analysis was performed according to the manufacturer’s instructions using 50 µl sample, standard, and buffer control. Samples were added into mixed Luminex array beads to incubation for 2 hours at room temperature. After washed, 25 µl detection antibody was added and incubated at room temperature for 30 min. After washed for 3 times, PE-Streptomyces anti-biotin protein (50µl) was added and incubated at room temperature for 10 min. After washed, 125µl test-buffer was added to re-suspend the beads and analyzed using Luminex machine 200.

### Statistical analysis

SPSS17.0 software was used to analyze the obtained data. Means±standard deviations are reported. Experiments were carried out independently three times. The two-sample t test was used for statistical analysis, and the survival rate was analyzed by log-rank test. P<0.05 was considered statistically significant.

## RESULTS

### High serum levels of soluble MICA are associated with poor HCC prognosis

**Figure 1.**
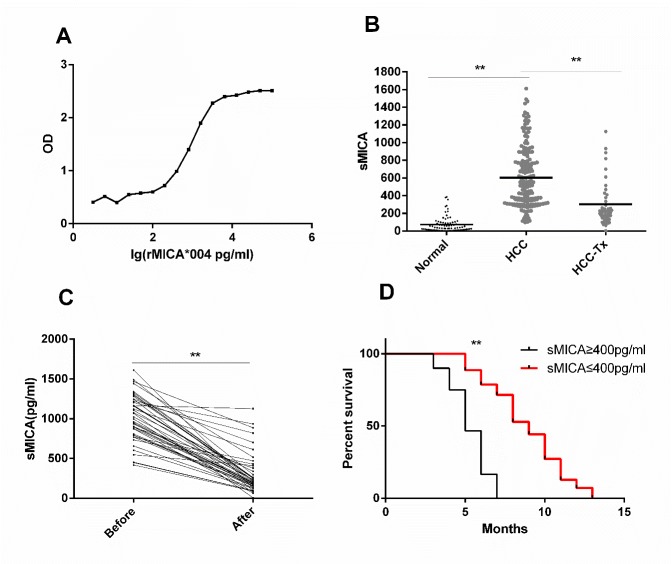
**Serum sMICA concentration is associated with poor prognosis in HCC**. **(A)** Standard curve for quantitative detection of sMICA using double-antibody sandwich ELISA made using purified rMICA*004 at eight concentrations ranging from 100 to 10000 pg/ml. Plotted is OD vs. lg[rMICA]. **(B)** sMICA concentrations (pg/ml) in serum of healthy controls (N=80), HCC patients (N=174), and HCC patients’ post-surgical resection of tumors (N=44). The horizontal lines indicate means. ** Indicates P<0.05. **(C)** Serum sMICA levels before and after surgery in HCC patients with serum sMICA ≥ 400 pg/ml before treatment (N=44). Serum samples were taken immediately prior to surgery and 15 days after surgery. Lines pair points from individual patients. ** Indicates P<0.05. **(D)** Survival curves of HCC patients not treated surgically divided based on serum sMICA concentration: sMICA^low^ < 400 pg/ml (N=70, red) and sMICA^high^ ≥ 400 pg/ml (N=60, black). Log-rank statistical analysis was performed on the survival curves between the two groups; ** Indicates P<0.05.

Increased release of soluble MICA into circulation is common in patients with solid tumors. In order to detect and analyze the presence of sMICA in peripheral blood of HCC patients, we established a dual-antibody sandwich ELISA method to quantitatively determine the concentration of sMICA in serum. A standard curve was prepared by analysis of known concentrations of highly purified recombinant (rMICA*004) (Fig. 1A). The range of sMICA concentration that could be accurately detected was 100 to 6309 pg/ml. Any serum samples that initially tested above 6309 pg/ml were retested after appropriate dilution.

The concentrations of sMICA in serum of healthy controls (N=80), HCC patients (N=174), and HCC patients’ post-surgery (N=44) were measured with the sandwich ELISA. The mean concentrations of serum sMICA were 72.26±87.88, 602.17±338.15, and 301.62± 288.20 pg/ml, respectively (Fig. 1B). The serum sMICA concentration of HCC patients was significantly higher than that of the controls (t=3.107, P=0.002). Of 80 HCC patients, 44 with serum sMICA concentrations that exceeded 400pg/ml were evaluated 15 days after resection of the liver tumor tissue. In these HCC patients, sMICA serum levels declined significantly post-surgery (1205.4± 740.5 pg/ml vs. 701.2±843.5 pg/ml, t=9.67, P<0.001), Furthermore, pair analysis of HCC cases before and after surgery showed that the concentration of serum sMICA decreased in each patient after the liver cancer tissue was removed (t=15.28, P < 0.001, Fig. 1C).

HCC patients who were not treated surgically were divided into two groups based on serum sMICA concentrations: the sMICA^high^ group (N=60) had sMICA levels greater than or equal to 400 pg/ml, and the sMICA^low^ group (N=70) had sMICA levels below 400 pg/ml. Mean survival of patients in sMICA^high^ group was shorter than that of patients in the sMICA^low^ group (5.3 months vs. 8.8 months, Fig. 1D). This difference was highly significant (χ^2^=81.988, P<0.001), suggesting that the concentration of sMICA in the blood is correlated with prognosis.

### Establishment of NKG2D signaling evaluation system

**Figure 2.**
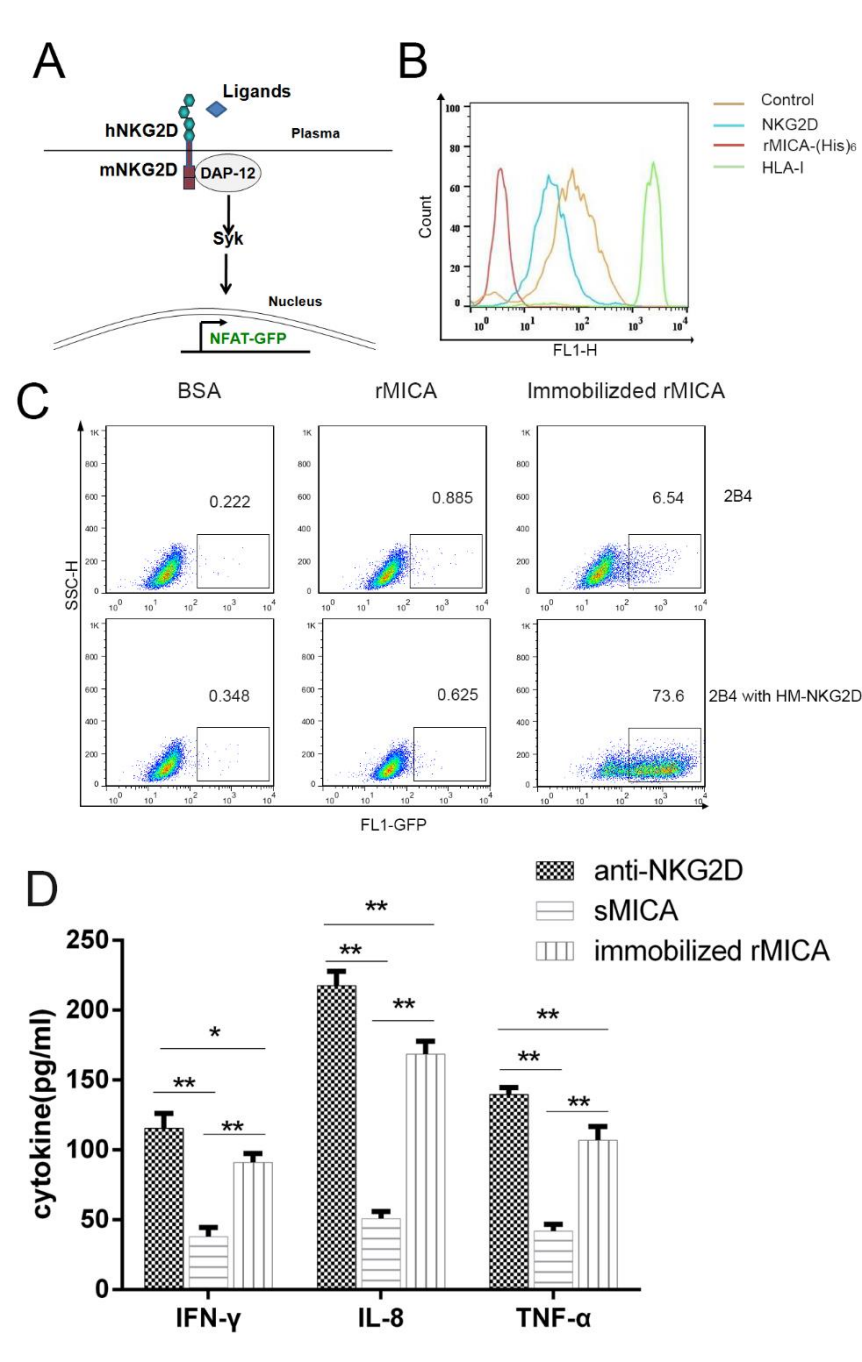
**sMICA does not activate the NKG2D receptor pathway**. **(A)** Diagram of NKG2D receptor reporter system. The NKG2D chimeric receptor is an engineered receptor composed of the human NKG2D extracellular domain and the mouse NKG2D membrane-spanning region. Upon binding of ligand to the chimeric NKG2D receptor, downstream signaling results in production of GFP. **(B)** Flow cytometry analysis of NKG2D-2B4 or control cells (2B4 cells) incubated with anti-NKG2D monoclonal antibody, anti-His tag monoclonal antibody, anti-HLA-I monoclonal antibody (w6/32), or mouse isotype IgG. **(C)** Percentage of GFP^+^ cells in NKG2D-2B4 and 2B4 cells incubated with BSA or soluble rMICA *004 or incubated in wells pre-coated with rMICA. **(D)** The cytokine responses of NK cells to anti-NKG2D monoclonal antibody coated (anti-NKG2D), soluble rMICA, and immobilized rMICA after 24 hours. The supernatants were collected and concentrations of IFN-γ, TNF-α, and IL-8 were determined (pg/ml). Plotted are results of triplicate experiments.

In order to determine whether sMICA influences signaling through the NKG2D receptor, we generated a combined NKG2D receptor cell model. We fused the extracellular domain of the human NKG2D to the transmembrane domain and cytoplasm tail of mouse NKG2D and expressed this construct in 2B4 cells (NKG2D-2B4 cells). This hybrid NKG2D receptor can bind NKG2D ligands (e.g., MICA) and activates signaling through the ITAM of the adapter membrane molecule DAP12 and the phosphorylation of Syk to activate transcription of GFP (Fig. 2A).

Since our data showed that the immobilized rMICA activated the NKG2D signaling pathway but not soluble sMICA, we used an anti-NKG2D monoclonal antibody to demonstrate the expression of NKG2D receptor on the NKG2D-2B4 cell surface and used an anti-His antibody to evaluate the binding of soluble MICA to the NKG2D-2B4 cells. High levels of NKG2D receptors were detected on the NKG2D-2B4 cell surface, and the soluble rMICA did bind to these cells (Fig. 2B). This suggests that although soluble rMICA did not activate NKG2D receptor-mediated signaling, it does bind to the NKG2D receptor.

After 24 hours of the incubation of 2B4 and NKG2D-2B4 cells with NKG2D ligands, flow cytometry was used to detect the percentage of GFP^+^ cells, which is indicative of ligand binding to the chimeric receptor and NKG2D pathway activation. In samples treated with rMICA*004 or with the control BSA, fewer than 1% of cells were GFP^+^ (Fig. 2C). The results showed that soluble rMICA*004 did not induce production of GFP. However, in cells incubated with immobilized rMICA, NKG2D receptor-mediated signaling was induced with 73.6% of cell expressing GFP. Less than 6.25% of 2B4 cells, which do not express the chimeric receptor but do encode GFP, were fluorescent after 24 hours (Fig. 2C; t=10.927, P < 0.001).

Activation of the NKG2D receptor pathway in NK cells should induce production of cytokines. Therefore, we measured cytokines released from NKL cultured in the presence of a monoclonal antibody, or soluble rMICA and immobilized-rMICA. After 24 hours, cell culture super-natants were collected and analyzed with the cytokine Luminex array. The amounts of IFN-γ, TNF-α, and IL-8 released upon stimulation with immobilized rMICA were significantly higher than levels in the presence of soluble rMICA (P < 0.001, Fig. 2D), and other 14 cytokines were not increased. Amounts of cytokines released from NKL cultured with immobilized rMICA were similar to amounts released in the presence of the positive control with anti-NKG2D monoclonal antibodies coated (Fig. 2D).

**Figure 3.**
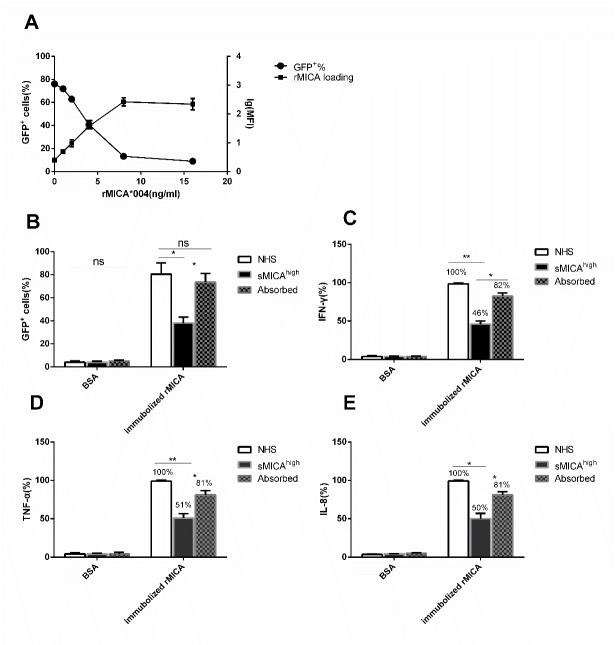
**Soluble MICA molecule blocks the activation of NKG2D pathway**. **(A)** Binding of soluble rMICA*004-His to NKG2D-2B4 cells detected by staining of the PE-conjugated anti-His-tag antibody. The log of mean fluorescence intensity (lg[MFI]) as a function of rMICA*004-His is plotted on the right-hand axis. The blocking effect on NKG2D pathway signaling was determined by analysis of GFP^+^ cells; the percent GFP^+^ cells in the presence of each concentration of rMICA*004-His is plotted on the left-hand axis. **(B)** Percentage of GFP^+^ cells in the presence of sMICA^high^ patient sera (N=10), the same sera pre-treated with anti-MICA mAb6B3 beads (Absorbed, N=10), and sera from healthy volunteers (NHS, N=10). * indicates statistically significant difference (P < 0.05). **(C-E)** Cytokines (C) IFN-γ, (D) TNF-α, and (E) IL-8 released from NKL incubated with immobilized rMICA. The concentrations of cytokines in supernatant of samples treated with NHS were defined as 100%. The ratio of sample treated with sMICA^high^ and Absorbed are given. * Indicates statistically significant difference (P < 0.05).

### sMICA blocks activation of NKG2D receptor-mediated signaling

An analysis of the geometrical mean of fluorescence intensity (lg[MFI]) was used to evaluate the effect of sMICA and immobilized rMICA on NKG2D report system. Binding increased as a function of soluble rMICA added (Fig. 3A). Conversely, the percentage of GFP^+^ cells decreased with increasing concentration of sMICA added (Fig. 3A). We then evaluated the effect of sera from sMICA^high^ HCC patients and found that addition of patient sera dramatically reduced the percentage of GFP^+^ cells compared to normal health serum (NHS, t=5.557, P=0.031, Fig. 3B). In the patient serum depleted of sMICA by absorption with an anti-MICA mAb6B3 beads, the percentage of GFP^+^ cells was near NHS control levels (t=5.433, p=0.032, Fig. 3B). These results support the hypothesis that sMICA inhibits activation of the NKG2D pathway.

We next evaluated cytokine release from cultured NKL incubated with immobilized-rMICA in presence of sera from healthy volunteers (NHS), sera from HCC patients wth high level of sMICA (sMICA^high^), sMICA^high^ sera depleted of sMICA (Absorbed). We found that IFN-γ, TNF-α, and IL-8 release was inhibited by in presence of sMICA compared to levels from cells cultured in the presence of NHS (Fig. 3C-E). Moreover, the effect was reversed when sMICA was removed from the serum by anti-MICA mAb6B3 beads (Fig. 3C-E).

**Figure 4.**
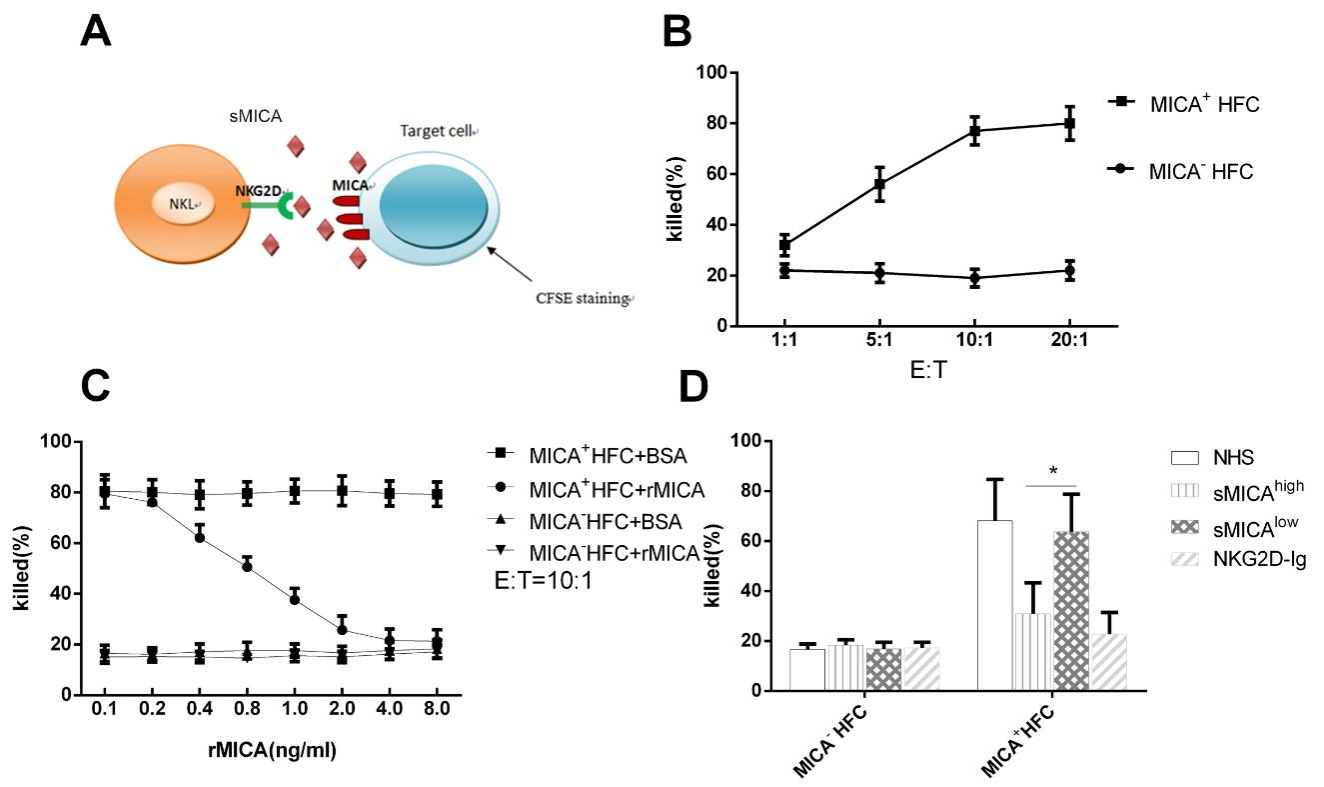
**sMICA inhibits the cytotoxicity of NK cells toward MICA^+^ target cells**. **(A)** Schematic of components involved in the MICA-NKG2D pathway. NK effector cells were incubated with MICA^+^ target cells stained with CSFE dye. sMICA interferes with cell killing mediated by NK cells. **(B)** Target cells (5000 cells per assay) with (MICA^+^ HFC) or without MICA expressed human fibroblasts (MICA^-^ HFC) were co-cultured with NKL at different E: T ratios. The mean percentages of dead cells from three replicate experiments are plotted. **(C)** The percent dead target cells in the presence of soluble rMICA, BSA at finally concentrations diluted from 0.1 to 8.0 ng/ml at E: T of 10:1. Plotted are means of triplicate experiments. **(D)** The percent dead target cells in the presence of serum from sMICA^high^ patients (N=10) and from sMICA^low^ patients (N=10) at E:T of 10:1. Normal health serum (NHS) was used as negative control and soluble NKG2D-Ig at concentration at 1.0 μg/ml were used as maximum blocking controls. Plotted are means of three experiments for each tested (C and D).

### sMICA reduces the cytotoxicity of NK cells

NK cells that express NKG2D receptors on the cell surface kill MICA-expressing target cells as we demonstrated previously [11]. To monitor the effect of sMICA on NK cell-mediate cytotoxicity, we incubated target cells, labeled with CSFE, with NKL, stained dead cells with 7-AAD dye, and analyzed the samples using by flow cytometry (Fig. 4A). As target cells, we used human fibroblasts engineered to stably express high levels of MICA*027 membrane proteins and fibroblasts that do not express MICA. The killing efficiency was measured at ratios of effector (NKL) to the target cells (E:T) of 1:1, 5:1, 10:1, and 20:1. Killing by NK cells was dependent on the NKG2D-MICA pathway as fibroblasts that do not express MICA were used as the control; the optimal E:T ratio was 10:1 (Fig. 4B).

Both soluble rMICA and BSA interfered with NK cell-mediated toxicity toward fibroblasts that express MICA in the immobilized rMICA stimulating system. In the presence of soluble rMICA and at an E: T of 10:1, we observed that the ratio of MICA+ target cell death were reduced from 79.5 % to 21.3 % as in the presence of the concentration of rMICA increased from 0.1 to 8.0 ng/ml, But with BSA concentration increased, the ratio of MICA^+^ target cells were remained at the level of 80% without any dropping. Using MICA^-^ HFC as target cells, there were only about 16% dead cells observed, in spite of in presence of rMICA or BSA in the same concentrations as above. (Fig. 4C). In a similar experiment, the maximum blocking effect of NKL on the cytotoxicity of MICA^+^ HFC cells was obtained in presence of NKG2D-Ig fusion protein at concentration above 1.0 μg/ml, and normal health serum used as negative blocking control (Fig. 4D). We tested the effects of sera from the sMICA^high^ and sMICA^low^ HCC patient groups on the killing of MICA^+^ HFC and MICA^-^ HFC by NKL in the immobilized rMICA stimulating system. At 1:2 dilution of sMICA^high^ sera (n=10), there were 30.8%±10.1% of the MICA-expressing target cells were killed compared with 63.7%±12.2% of the target cells killed in presence of 1: 2 dilution of sMICA^low^ sera (n=10) from HCC (Fig. 4D); More cells were escaped from killing in presence of sMICA^high^ serum than that of sMICA^low^ serum (t=2.919, P=0.043). There was no difference observed when used MICA^-^ HFC as the target cells, suggesting that high level of sMICA in serum of HCC patient might protect hepatocellular carcinoma from host immune response.

## DISCUSSION

HCC is the most common primary liver cancer, accounting for 90% of all liver cancers. The tumor cells escape immune response by T lymphocytes and NK cells in various ways [30, 31]. Recent studies have shown that the mechanisms of immune escape involve activation of immunosuppressive receptors that negatively regulate the immune response and down-regulate host immune clearance of cancerous tissues [32] and the inhibition of activating receptors generally reduces the immune response [33]. Activation receptor NKG2D on NK and T cells is involved in anti-tumor immune responses [34]. Here we showed that a high level of soluble sMICA, an NKG2D ligand, in sera of HCC patients was associated with poor outcome. Wan and his team [35] reported that MICA is released into circulation due to shedding of MICA from cell membranes during tumor transformation or upon stress. We showed that sMICA binds to cells expressing the NKG2D receptor but does not activate NKG2D receptor-mediated signaling and does not activate of NK cells [25]. It was previously reported that the binding of sMICA decreases the expression of NKG2D receptor in NK cells [36]. Zou and co-workers reported that high level of serum sMICA is associated with biliary cast syndrome after liver transplantation [37].

Using NKG2D reporter cells, we showed that the soluble rMICA did not activate the NKG2D pathway, although it bound to the NKG2D-2B4 reporter cells. In contrast, when we incubated the NKG2D-2B4 reporter cells with immobilized rMICA, 73.6% of cells fluoresced, indicating active NKG2D signaling, after 16 hours of culture. We also demonstrated that NKG2D pathway activation in NK cell lines with immobilized rMICA, but this activation of NKG2D could be blocked by soluble recombinant MICA molecules and by serum from HCC patients that expressed high levels of soluble MICA. Interestingly, immobilized rMICA also induced release of cytokines from NKL cells. These experiments are used as the well-model to understand how sMICA inhibits the immuno-response of NK and CD8+ T cells to tumor cells. Using microscopy, we previously observed that the single NKG2D dimer is not activated until multiple NKG2D dimers gather into a bundle [25]. This aggregation promotes DAP12 phosphorylation and downstream signaling. Soluble MICA does not induce NKG2D receptor aggregation and there is nothing of NKG2D signaling. The performance of this engineering receptor of NKG2D was confirmed by the natural NKG2D receptor of NKL cells.

Since NKG2D receptor is composed of two identical NKG2D molecules, multiple NKG2D receptors might be formed into agglomeration on cell surface when NKG2DLs bind. The immobilized rMICAs can bind and aggregate NKG2D receptors. Therefore, culturing NK cells with immobilized initiates NKG2D receptor pathway. In the study, we are first to use the immobilized rMICA to activate NK cell lines, instead of using MICA-expressing cells. Cytokines released by NKL reflected NKL activation. We found that IFN-γ, TNF-α and IL-8 were released increasely when NKLs were stimulated by the immobilized rMICA. Our results demonstrated the blocking effect of sMICA on NKL activation in both fluorescence reporter system and cytokine releasing.

In order to further validate that serum sMICA blocks killing of tumor cells by NK cells, we used human fibroblasts that stably express MICA as the target cells. In the absence of added sMICA, about 80% of MICA-expressing fibroblasts were killed at an E:T of 10:1. However, in presence of sMICA added, either the soluble rMICA or soluble MICA in sera of sMICA^high^ HCC patient, the killing of fibroblasts that express MICA was significantly decreased. Since the test for NKG2D pathway activation are based on the immobilized rMICA stimulation, using MICA-expressed cells as the target may be complicate because of many membrane proteins on cell surface. The inhibition of NK cytotoxicity to the target cells can be reversed when serum sMICA is removed (Fig. 3C-E).

It was previously reported that the formation of sMICA is associated with the MICA*008 allele [38], which produces a truncated protein. This short MICA molecule may be more readily released by exosomes from the cell membrane than the wild-type version [39], most other allelic MICA variants are released by proteolytic shedding [18, 19]. No such association with MICA*008 mutations were observed in our cohort (data not shown). A limitation of this study was that we did not evaluate other NKG2D ligands, such as sMICB and sULBP, which are potentially present in serum of HCC patients [40, 41]. In a future study, we will screen for all known soluble NKG2D ligands in sera of HCC patients.

In conclusion, this study demonstrated that high levels of serum MICA are correlated with poor prognosis in HCC cases. Our in vitro and cell-based experiments suggest that soluble MICA mediates tumor immune escape through blocking of the NKG2D signaling pathway. This analysis first demonstrated that the blocking effect of serum sMICA on the NKG2D pathway activation. Our findings indicate that sMICA potentially functions as a biomarker for identification of HCC patients at high risk, and also can provide a novel approach for tumor immunotherapy.
